# *Lactobacillus acidophilus* ATCC 4356 Alleviates Renal Ischemia–Reperfusion Injury Through Antioxidant Stress and Anti-inflammatory Responses and Improves Intestinal Microbial Distribution

**DOI:** 10.3389/fnut.2021.667695

**Published:** 2021-05-11

**Authors:** Peng Zhang, Xiuwu Han, Xin Zhang, Xuhui Zhu

**Affiliations:** Department of Urology, Beijing Chao-Yang Hospital, Capital Medical University, Beijing, China

**Keywords:** *Lactobacillus acidophilus* (ATCC 4356), renal ischemia-reperfusion injury, intestinal microbial, antioxidant stress, anti-inflammatory

## Abstract

**Background:** Ischemia–reperfusion injury (IRI) is one of the main causes of acute kidney injury. Our previous results have shown that anti-oxidative stress decreased in the renal IRI model. This study aimed to investigate the effect of *Lactobacillus acidophilus* ATCC 4356 on oxidative stress, inflammation, and intestinal flora in renal IRI.

**Methods:** The model of renal IRI was established by cross-clamping the renal pedicle with non-traumatic vascular forceps. H&E staining was applied to observe the damage of kidney tissue in each group. The concentrations of serum blood urea nitrogen (BUN), creatinine (Cre), superoxide dismutase (SOD), glutathione (GSH), and malondialdehyde (MDA) were detected by biochemical kit. ELISA measured the concentrations of interleukin (IL)-1β, IL-8, IL-4, and IL-10. qRT-PCR was performed to detect molecular expressions of ATCC 4356, oxidative stress-related factors [nuclear factor-related factor 2 (Nrf2), heme oxygenase 1 (HO-1)], inflammatory factors [tumor necrosis factor (TNF)-α, IL-1β, IL-8, interferon (IFN)-γ, IL-4, IL-10], and apoptosis-related factors [caspase 3, Bax, Bcl2, high-mobility group box protein 1 (HMGB1)]. Except for ATCC 4356, the protein expression of the above indicators was detected by Western blot. The apoptosis level of renal tissue cells was detected by TdT-mediated dUTP nick end labeling (TUNEL). 16S rDNA gene sequencing was used to detect the changes of microbial species in the contents of the duodenum and screen out the differentially expressed flora.

**Results:** Both the glomeruli and renal tubules of ischemia/reperfusion (I/R) mice were severely damaged. H&E result displayed that *L. acidophilus* ATCC 4356 attenuated the infiltration of inflammatory cells caused by I/R. ATCC 4356 reduced the high expression of BUN and Cre in I/R mice with a dose effect. It also reduced the high expression of MDA, TNF-α, IL-1β, IL-8, IFN-γ, caspase 3, Bax, and HMGB1 in I/R mice, while it increased the low expression of SOD, GSH, Nrf2, HO-1, IL-4, IL-10, and Bcl2 in I/R mice. ATCC 4356 inhibited the high level of apoptosis in the kidney tissue of I/R mice. In IRI mice, the top 3 different gut microbiota were *Helicobacter, cultivated_bacterium*, and *k__Bacteria_ASV_3* compared with sham mice. Oral *L. acidophilus* ATCC 4356 reversed this change.

**Conclusion:** *L. acidophilus* ATCC 4356 attenuated renal IRI through anti-oxidative stress and anti-inflammatory response and improved the intestinal microbial distribution.

## Introduction

Ischemia–reperfusion injury (IRI) is one of the main causes of acute kidney injury (AKI), which usually occurs during renal surgery (1). Renal IRI is a major clinical challenge faced by clinicians during the operation period of renal transplantation (2). Renal IRI is associated with high morbidity and mortality, and the pathophysiological process is complicated, while there is no good treatment method (3).

Oxidative stress, inflammation, and apoptosis are not only important causes of renal IRI but also key factors that cause renal insufficiency (4). For example, oxidative stress, inflammation, and apoptosis in diabetic rat models are intensified, thereby exacerbating rat renal IRI (5). Studies have found that resveratrol (RSV) decreased oxidative stress and inhibited inflammatory responses, which played a role in kidney protection (6). In addition, fibroblast growth factor 10 (FGF10) prevented renal IRI by regulating autophagy and inflammatory signal transduction (7). Similarly, nobiletin inhibited inflammatory cytokines and regulated inducible nitric oxide synthase (iNOS)–endothelial nitric oxide synthase (eNOS) expression, thereby protecting rats from renal IRI (8). Gastrin attenuated renal IRI by anti-apoptosis (9). Congruously, our previous results have indicated that anti-oxidative stress could alleviate renal IRI. Therefore, it may be a feasible way to prevent or reduce renal IRI by inhibiting oxidative stress, inflammation, and apoptosis.

Importantly, oral *Lactobacillus acidophilus* has the effect of anti-inflammation, anti-oxidative stress, and regulating intestinal microflora homeostasis, thus contributing to health benefits (10). In terms of antioxidant stress, oral *L. acidophilus* ATCC 4356 relieved the process of atherosclerosis by anti-oxidative stress (11). Oral *L. acidophilus* ATCC 4356 alleviated diabetic complications by antioxidant stress (12). In terms of anti-inflammation, oral *L. acidophilus* attenuated traumatic brain injury by anti-inflammatory response (13). We suspected that *L. acidophilus* ATCC 4356 was likely to exert an effect on renal IRI by regulating oxidative stress and inflammation.

A growing body of evidence has shown that the intestinal flora plays an important role in health and disease by regulating local and systemic immunity. Effective interventions of probiotic supplements on the composition of the intestinal flora can improve health and prevent the onset of certain diseases. For example, oral *L. acidophilus* reduced bacterial translocation and liver cell damage by regulating intestinal flora (14). We speculated that *L. acidophilus* ATCC 4356 alleviated renal IRI by regulating intestinal flora. In mechanism, the intestinal flora reached two sites [kidney and bone marrow (BM)] at the same time due to circulation. On the one hand, it reduced the maturation state of macrophages/monocytes. On the other hand, it inhibited the release of chemokines [monocyte chemoattractant protein (MCP)-1 and macrophage inflammatory protein (MIP)-2α] and the main functions (migration capacity). This reduced the influx of granulocytes to protect the kidney from damage (15).

In summary, this study will explore the effect of *L. acidophilus* ATCC 4356 on oxidative stress, inflammation, and intestinal flora in the renal IRI model. Our findings may provide a new prevention and treatment strategy for renal IRI diseases.

## Materials and Methods

### Renal Ischemia/Reperfusion Model Construction

Eight-week-old specific pathogen-free (SPF)-grade C57/BL6 male mice were housed in standard laboratory cages and allowed free access to food and water. All experimental protocols were approved by the Animal Ethics Committee of Capital Medical University. The mice were anesthetized by intraperitoneal injection of sodium pentobarbital (50 mg/kg body weight). It was then placed on a heating pad to maintain the body temperature at 37°C. Laparotomy was performed on the animal. The renal hilum was exposed bilaterally. The bilateral renal pedicle was cross-clamped with non-traumatic vascular forceps for 28 min to complete renal ischemia. Before the end of the ischemic period, the cross-clamped with non-traumatic vascular forceps were removed. Renal ischemia during clamping and subsequent renal reperfusion after release of clamping were visually monitored by renal discoloration and recoloration, respectively. The bilateral kidney was observed for 5 min to ensure reperfusion for 48 h. The animals in the Sham group underwent the same operation without clamping the kidney pedicle. The abdomen was sutured with 5.0 Monocryl sutures (Ethicon, USA).

### Preparation of *Lactobacillus acidophilus* ATCC 4356

*L. acidophilus* ATCC 4356 was obtained from ATCC (Manassas, Virginia, USA). The original culture was stored in 40% (volume/volume) glycerin at −80°C prior to use. The 1% inoculum was grown in sterile De Man, Rogosa, and Sharpe broth (DIFCO, Detroit, Michigan, USA). The organisms were subcultured three times and then grown at 37°C for 16 h. The inoculum was stored at 4°C between transfers.

### Lactobacillus Acidophilus ATCC 4356 Intervention

Sixty 8-week-old SPF C57/BL6 male mice were randomly divided into five groups (n = 10). The 40 mice were established with renal IRI model. Model mice were given vehicle, 1 ^*^ 10^8^ CFU/ml, 5 ^*^ 10^8^ CFU/ml, 1 ^*^ 10^9^ CFU/ml *L. acidophilus* ATCC 4356 by intragastric gavage, 0.2 ml/head/day for 4 weeks, respectively. The remaining 10 mice were in the Sham group, which were given an equal-volume vehicle. The experiment was divided into Sham group, ischemia/reperfusion (I/R) group, La.L group, La.M group, and La.H group. At the fourth weekend of the intervention, all animals were sacrificed. Samples needed for testing were collected.

### Hematoxylin–Eosin Staining

The mouse kidney tissue was fixed in 4% paraformaldehyde for more than 24 h. The tissue was flushed with running water. The tissue was dehydrated by gradient ethanol and was transparent by xylene. Subsequently, the tissue was embedded in paraffin. A paraffin microtome (YD-315, Yidi, China) was used to prepare 4-μm-thick sections. The slices were baked in a 62°C oven for more than 8 h. The tissue was deparaffinized and rehydrated with xylene and gradient ethanol. The cytoplasm was stained with eosin to varying degrees of pink or red, in sharp contrast to the blue nucleus stained with hematoxylin. The sections were observed under an optical microscope (BA210T; Motic, Singapore).

### Biochemical Testing

Experimental procedures were strictly performed according to the biochemical kit (C013-1, C011-2-1, A001-3, A006-2-1, A003-1-2; Nanjing Jiancheng Bioengineering Institute, China). The absorbance values of each group at 640-, 546-, 450-, 405-, and 532-nm wavelengths were detected using a microplate reader (MB-530, Huisong, China). The contents of blood urea nitrogen (BUN), creatinine (Cre), superoxide dismutase (SOD), glutathione (GSH), and malondialdehyde (MDA) in serum were calculated through the formula.

### ELISA

The blood was centrifuged at 1,000g for 20 min at 2–8°C. The supernatant was collected. ELISA kits (CSB-E08054m, CSB-E04634m, CSB-E04594m; Wuhan Huamei, China) and (ml063162; Shanghai Meilian, China) were used to detect the concentrations of interleukin (IL)-1β, IL-4, IL-10, and IL-8. The experimental instructions were strictly implemented. The absorbance values of each group at 450-nm wavelength were detected through the microplate reader.

### Quantitative Real-Time Polymerase Chain Reaction

Total RNA was isolated from kidney tissues in each group using TRIzol^®^ reagent (Thermo Fisher, 15596026, USA). The cDNAs were synthesized using mRNA reverse transcription kit (CW2569, Kangwei reagent, China). UltraSYBR Mixture (CW2601, Kangwei Reagent, China) was used for PCR reaction. The fluorescent quantitative PCR system was ThermoFisher (PikoReal 96). Glyceraldehyde 3-phosphate dehydrogenase (GAPDH) was used as an internal reference. The relative expression level was calculated using the 2^−ΔΔCt^ method. The sequences of mRNA primers are in Table 1.

**Table 1:** Primer sequences.

**Name**	**Sequences**
Nrf2	Forward GCTCCTATGCGTGAATCCCAA Reverse TTTGCCCTAAGCTCATCTCGT
HO-1	Forward TCCATGTTGACTGACCACGACT Reverse CCCACCCCTCAAAAGATAGCC
TNF-α	Forward AGCACAGAAAGCATGATCCG Reverse CACCCCGAAGTTCAGTAGACA
IL-1β	Forward TGAAATGCCACCTTTTGACAGT Reverse TTCTCCACAGCCACAATGAGT
IL-8	Forward AGACAGAGATACCGCCACGTTC Reverse AGAGAAAGCCTACACACAGTCCT
IFN-γ	Forward GCCACGGCACAGTCATTGA Reverse TGCTGATGGCCTGATTGTCTT
IL-4	Forward ATGTACCAGGAGCCATATCCACGG Reverse TCCCTTCTCCTGTGACCTCGTT
IL-10	Forward GTTCCCCTACTGTCATCCCC Reverse AGGCAGACAAACAATACACCA
Caspase 3	Forward TCTGACTGGAAAGCCGAAACTCT Reverse AGCCATCTCCTCATCAGTCCCA
Bax	Forward TGAAGACAGGGGCCTTTTTG Reverse AATTCGCCGGAGACACTCG
Bcl2	Forward TTGAAAACCGAACCAGGAATTGC Reverse GTCCTGTGCCACTTGCTCT
HMGB1	Forward ATCGTTCTCTTAAAGTGCCAGT Reverse ACGCAAATGTAAAGAACCCAAG
GAPDH	Forward GCGACTTCAACAGCAACTCCC Reverse CACCCTGTTGCTGTAGCCGTA

*GAPDH, glyceraldehyde 3-phosphate dehydrogenase; HMGB1, high-mobility group box protein 1; HO-1, heme oxygenase 1; IFN-γ, interferon-γ; IL-4, interferon; Nrf2, nuclear factor-related factor 2; TNF-α, tumor necrosis factor-α*.

### Bacterial Quantitative Real-Time Polymerase Chain Reaction

According to the manufacturer's instructions, the QIAamp DNA Fecal Mini Kit (Qiagen, Hilden, Germany) was used to extract bacterial DNA from the digestion of the colon of C57/BL6 mice. UltraSYBR Mixture (CW2601, Kangwei Reagent, China) was used for PCR reaction. The fluorescent quantitative PCR system was ThermoFisher (PikoReal 96). The primers to quantify *L. acidophilus* ATCC 4356 are listed in Table 2. The initial DNA denaturation step was at 95°C for 10 min. Thirty amplification cycles (95°C for 15 s, 55°C for 25 s, and 72°C for 20 s) were performed. The Cp-value was drawn by using the DNA ASSAY kit (Qiagen, Hilden, Germany). The standard curve was drawn. Real-time monitoring was achieved by measuring fluorescence at the end of the extension phase.

**Table 2:** Sequence of primers used for detection of bacteria.

**Target**	**Sequence**
Sequences of primers used for detection of bacteria	Forward CTTCGGTGATGACGTTGGGA Reverse CTTCGGTGATGACGTTGGGA

### Western Blot

The kidney tissues of each group were taken out at −80°C. Appropriate amount of radioimmunoprecipitation assay (RIPA) lysis buffer (P0013B; Shanghai Biyuntian, China) was added to lyse the samples. The cell supernatant was collected through centrifugation. The instructions of the bicinchoninic acid (BCA) protein quantitative kit were strictly implemented to determine the protein concentration. Next, we took the same mass of protein and loaded it on the Bolt Bis-Tris gel. After electrophoresis, the protein was transferred to the membrane. The membrane was immersed in 5% skimmed milk powder and sealed at room temperature for 1 h. The sample was incubated with an appropriate amount of primary antibody at room temperature for 90 min, including Nrf2 (16396-1-AP, 1:1,000; Proteintech, USA), heme oxygenase 1 (HO-1; 10701-1-AP, 1:3,000; Proteintech, USA), tumor necrosis factor (TNF)-α (ab6671, 1:2,000; Abcam, UK), IL-1β (16806-1-AP, 1:2,000; Proteintech, USA), IL-8 (ab10727, 1:1,000; Abcam, UK), interferon (IFN)-γ (15365-1-AP, 1:2,000; Proteintech, USA), IL-4 (ab239508, 1:5,000; Abcam, UK), IL-10 (ab133575, 1:1,000; Abcam, UK), caspase 3 (19677-1-AP, 1:2,000; Proteintech, USA), Bax (50599-2-lg, 1:6,000; Proteintech, USA), Bcl2 (12789-1-AP, 1:6,000; Proteintech, USA), high-mobility group box protein 1 (HMGB1; 10829-1-AP, 1:1,500; Proteintech, USA), and internal reference β-actin (60008-1-Ig, 1:5,000; Proteintech, USA). The samples were incubated with secondary antibody horseradish peroxidase (HRP)-goat anti-rabbit IgG (SA00001-2, 1:6,000; Proteintech, USA) or HRP goat anti-mouse IgG (SA00001-1, 1:5,000; Proteintech, USA) at room temperature for 90 min. The sample was exposed to enhanced chemiluminescence (ECL) development.

### TdT-Mediated dUTP Nick End Labeling

Mouse kidney tissue was fixed in 4% paraformaldehyde for more than 24 h. The tissue was dehydrated by gradient ethanol and was transparent by xylene. Subsequently, the tissue was embedded in paraffin. A paraffin microtome (YD-315, Yidi, China) was used to prepare 4-μm-thick sections. The slices were baked in a 62°C oven for more than 8 h. The tissue was deparaffinized and rehydrated with xylene and gradient ethanol. The instructions of TdT-mediated dUTP nick end labeling (TUNEL) kit (40306ES50, Yeasen, China) were strictly carried out. The apoptosis of kidney tissues in each group was observed under a fluorescence microscope (BA410T; Motic, Singapore). Here, 3–5 400× visual fields for each group were randomly selected. Apoptosis rate (number of positive nuclei under the field of view/total number of nuclei under the field of view) was evaluated in each group.

### 16s rDNA

Based on the manufacturer's recommendations, microbial genomic DNA was extracted from the duodenal contents using the QIAamp^®^ Fast DNA Stool Mini Kit (QIAGEN). The quality of the extracted DNA was detected using the Agilent 4200 Tapestation (Agilent Technologies) Kit. The NextEra XT DNA Sample Prep Kit (Illumina) was used to generate the sequencing library. The Agilent 4200 Tapestation confirmed the quality of the library. The whole genome of the samples was sequenced on HiSeq 2500 platform (Illumina) to obtain the original data for quality control.

### Data Analysis

All data are expressed as mean ± standard deviation. All experiments were repeated three times independently. GraphPad Prism 8.0 statistical software was used to compare the data between two or three groups using Student's *T*-test or one-way analysis of variance. P < 0.05 was considered statistically significant.

## Results

### *Lactobacillus acidophilus* ATCC 4356 Relieved Renal Injury in Mice With Ischemia/Reperfusion

**Figure 1:**
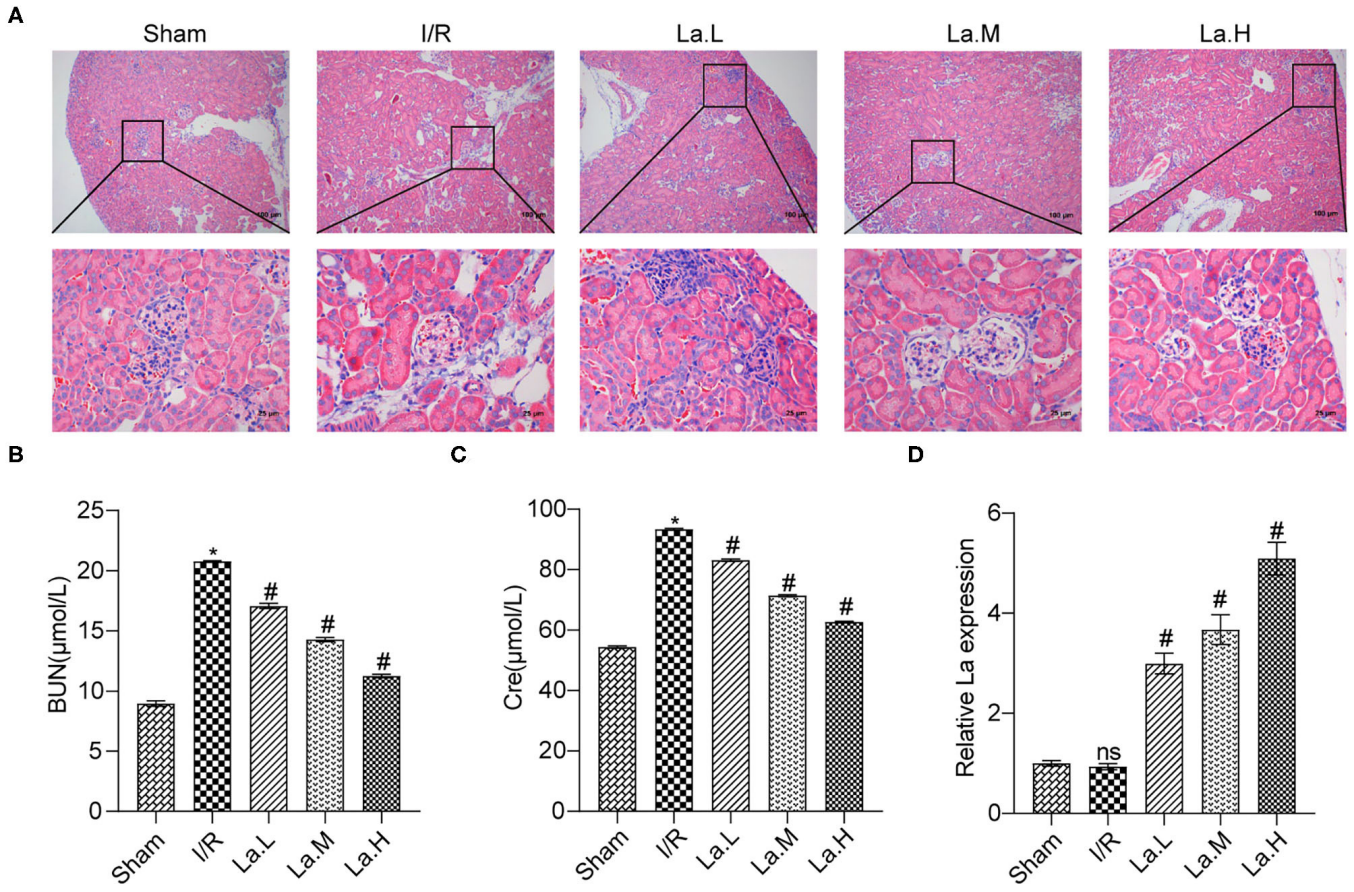
*Lactobacillus acidophilus* ATCC 4356 relieved renal injury in mice with ischemia/reperfusion (I/R). **(A)** The kidney tissue damage was observed by H&E staining in each group. **(B)** *L. acidophilus* ATCC 4356 reduced serum blood urea nitrogen (BUN) expression in I/R mice. **(C)** *L. acidophilus* ATCC 4356 decreased serum creatinine (CRE) expression in I/R mice. **(D)** *L. acidophilus* ATCC 4356 was colonized in the intestine of I/R mice. All data are expressed as mean ± standard deviation. All experiments were repeated three times independently. Statistical significance was calculated using one-way analysis of variance. *p < 0.05 vs. Sham, ^#^*p* < 0.05 vs. I/R.

H&E results showed that the I/R model caused serious damage to the glomerulus and renal tubules, and a large number of inflammatory cell infiltrates was seen in the renal tissue. *L. acidophilus* ATCC 4356 attenuated the damage and the inflammatory cell infiltration caused by I/R (Figure 1A). ELISA was applied to detect the changes of serum urea nitrogen (BUN) and Cre in each group. Renal IRI caused a significant increase in the expression of BUN and Cre in serum, while ATCC 4356 decreased the expression of BUN and Cre (Figures 1B,C). qRT-PCR was used to detect the colonization of ATCC 4356 in the colon. The results showed that there were a certain number of copies of ATCC 4356 in I/R mice (Figure 1D). The effect of ATCC 4356 showed a dose effect. Therefore, ATCC 4356 (1 × 10^9^ CFU/ml) was selected for the follow-up study. In summary, *L. acidophilus* ATCC 4356 alleviated renal injury in mice with I/R.

### *Lactobacillus acidophilus* ATCC 4356 Attenuated the Level of Oxidative Stress in Mice With Renal Ischemia–Reperfusion Injury

**Figure 2:**
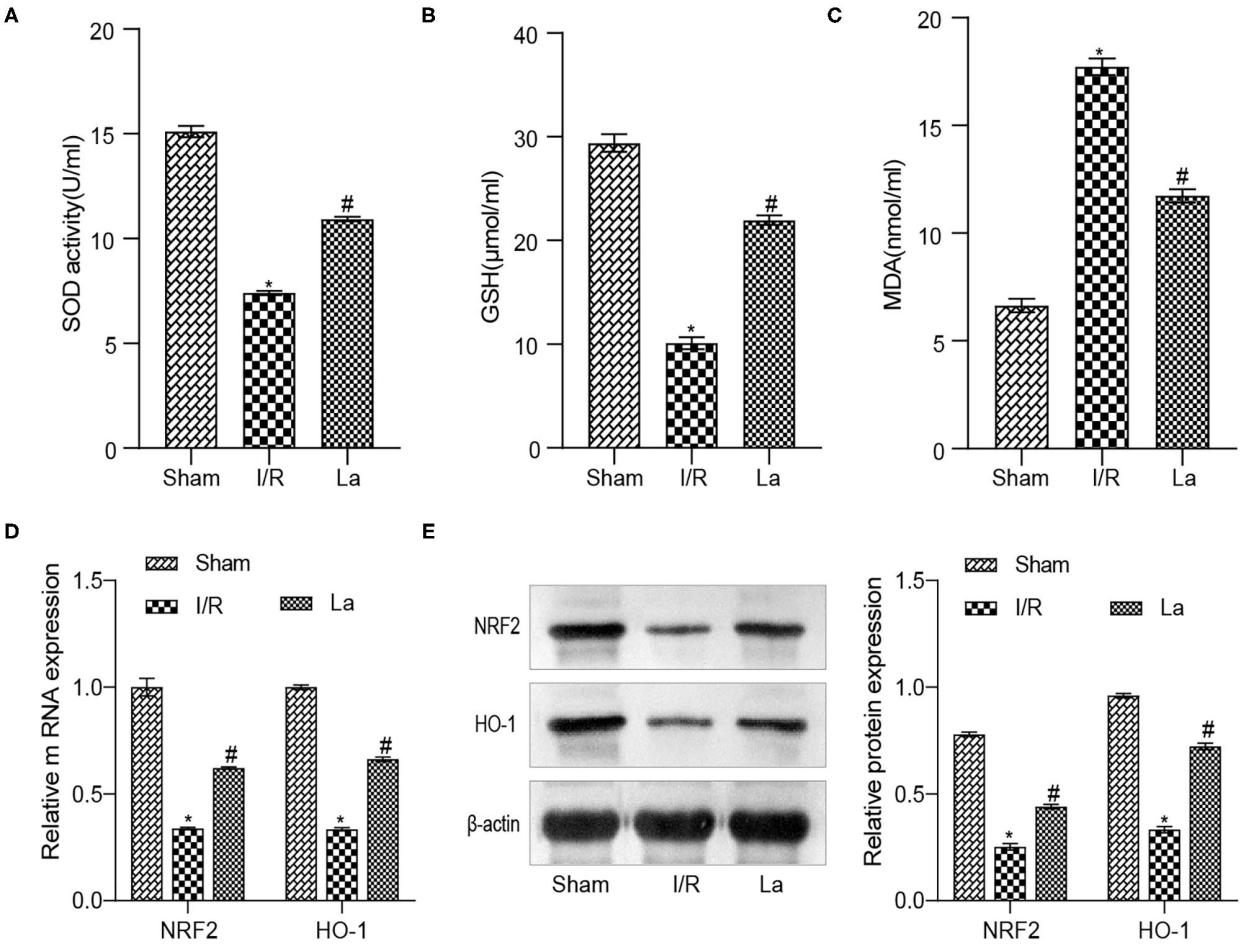
*Lactobacillus acidophilus* ATCC 4356 reduced the level of oxidative stress in mice with renal ischemia–reperfusion injury (IRI). **(A)** ATCC 4356 elevated superoxide dismutase (SOD) concentration in ischemia/reperfusion (I/R) mice. **(B)** ATCC 4356 promoted glutathione (GSH) expression in I/R mice. **(C)** ATCC 4356 decreased malondialdehyde (MDA) levels in I/R mice. **(D)** ATCC 4356 promoted the expression of nuclear factor-related factor 2 (Nrf2) and heme oxygenase 1 (HO-1) mRNA in I/R mice. **(E)** ATCC 4356 increased Nrf2 and HO-1 protein expression in I/R mice. All data are expressed as mean ± standard deviation. All experiments were repeated three times independently. Statistical significance was calculated using one-way analysis of variance. *p < 0.05 vs. Sham, ^#^*p* < 0.05 vs. I/R.

The Nrf2/antioxidant responsive element (ARE) signaling pathway is a key pathway in the anti-oxidative damage. Our previous experimental results proved that the expression of Nrf2 and HO-1 decreased in the renal IRI model, which indicated that the level of oxidative stress elevated. To determine the effect of ATCC 4356 on oxidative stress levels in mice with renal IRI, we used ELISA to detect the expression of SOD, GSH, and MDA in serum. Compared with the Sham group, the levels of SOD and GSH in the I/R group decreased, and the levels of MDA increased. ATCC 4356 elevated the expression of SOD (Figure 2A) and GSH (Figure 2B) in I/R mice, while it decreased the expression of MDA (Figure 2C) in I/R mice. The expression of Nrf2 and HO-1 molecules and protein in kidney tissue were detected by qRT-PCR and Western blot. ATCC 4356 increased the levels of Nrf2 and HO-1 molecules in the kidney tissue of I/R mice (Figure 2D). The protein level and molecular level of the above two indicators were consistent (Figure 2E). *L. acidophilus* ATCC 4356 reduced the level of oxidative stress in mice with renal IRI.

### *Lactobacillus acidophilus* ATCC 4356 Inhibited the Expression of Inflammatory Factors in Mice With Renal Ischemia–Reperfusion Injury

**Figure 3:**
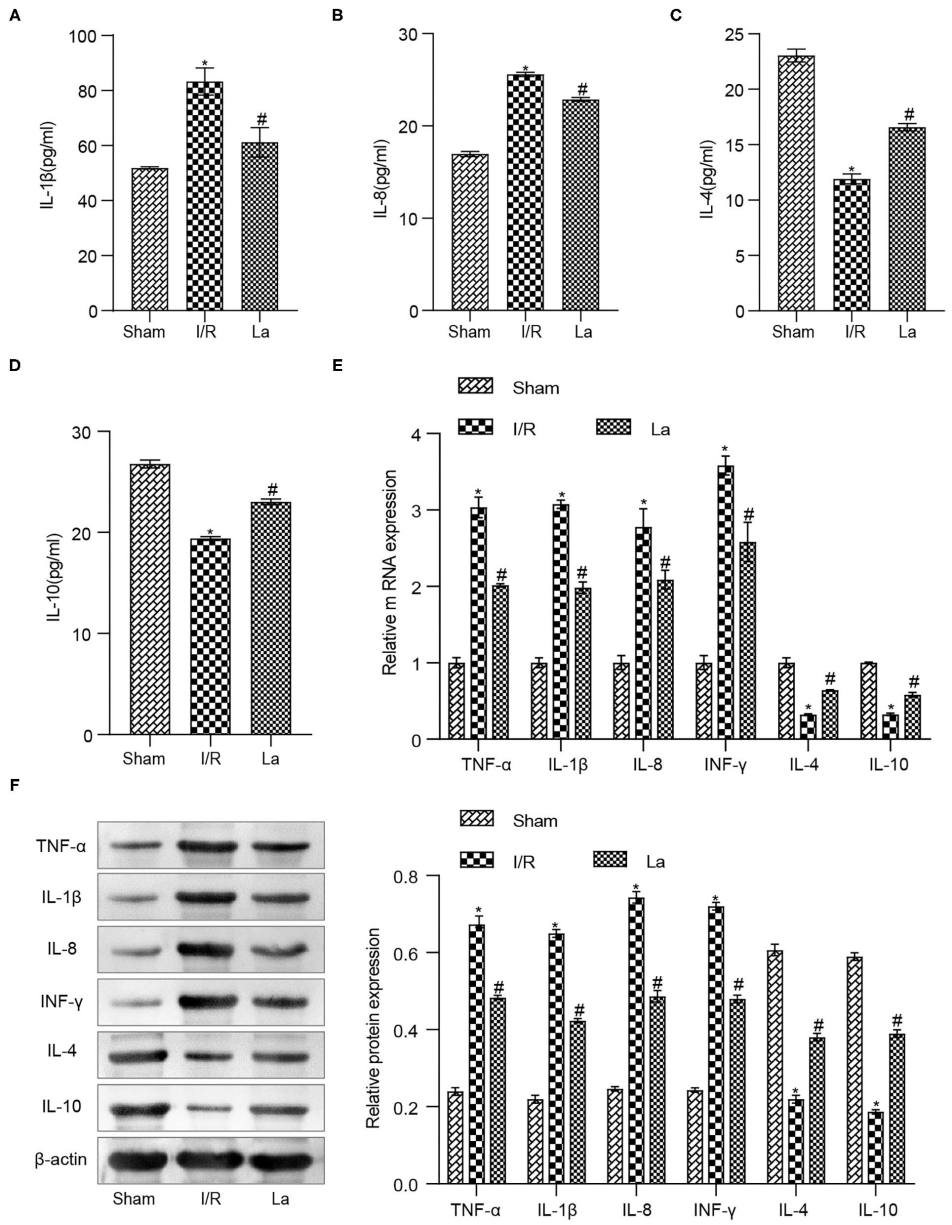
*Lactobacillus acidophilus* ATCC 4356 inhibited the expression of inflammatory factors in mice with renal ischemia–reperfusion injury (IRI). **(A)** Interleukin (IL)-1β concentration in serum. **(B)** IL-8 concentration in serum. **(C)** IL-4 concentration in serum. **(D)** IL-10 concentration in serum. **(E)** Molecular levels of tumor necrosis factor (TNF)-α, IL-1β, IL-8, interferon (IFN)-γ, IL-4, and IL-10 in kidney tissue. **(F)** Protein levels of TNF-α, IL-1β, IL-8, IFN-γ, IL-4, and IL-10 in kidney tissue. All data are expressed as mean ± standard deviation. All experiments were repeated three times independently. Statistical significance was calculated using one-way analysis of variance. *p < 0.05 vs. Sham, ^#^*p* < 0.05 vs. I/R.

We have previously confirmed that *L. acidophilus* ATCC 4356 could alleviate renal IRI. Renal IRI is often accompanied by inflammatory response. Based on this, we examined inflammatory factors in mice with renal IRI. The results of the ELISA experiment showed that ATCC 4356 reduced the expression of pro-inflammatory factors (IL-1β and IL-8) in the serum of I/R mice, while it elevated the expression of anti-inflammatory factors (IL-4 and IL-10) (Figures 3A–D). The above indicator expressions in the kidney tissue were also obtained by qRT-PCR and Western blot experimental methods, and the expression trend was consistent with that in ELISA. In addition, ATCC 4356 inhibited the high expression of TNF-α and IFN-γ in kidney tissue induced by I/R significantly (Figures 3E,F).

### *Lactobacillus acidophilus* ATCC 4356 Inhibited Cell Apoptosis in Mice With Renal Ischemia–Reperfusion Injury

**Figure 4:**
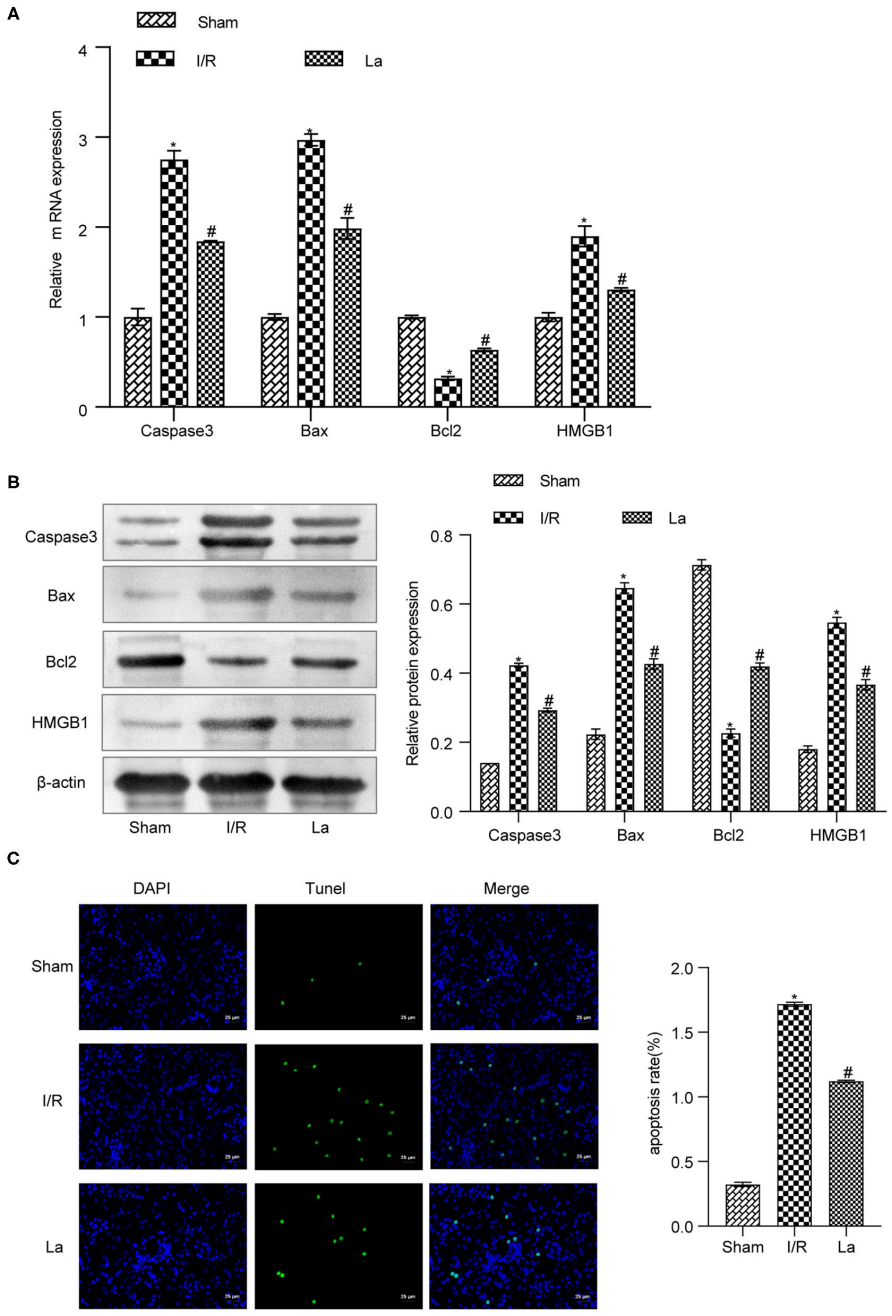
*Lactobacillus acidophilus* ATCC 4356 inhibited cell apoptosis in mice with renal ischemia–reperfusion injury (IRI). **(A)** Relative mRNA levels of caspase 3, Bax, Bcl2, and high-mobility group box protein 1 (HMGB1) in renal tissues of each group. **(B)** The relative protein expression of caspase 3, Bax, Bcl2, and HMGB1 in kidney tissues of each group. **(C)** TdT-mediated dUTP nick end labeling (TUNEL; 400×, scale bar = 25 μm) to evaluate the apoptosis level of renal tissue in each group. All data are expressed as mean ± standard deviation. All experiments were repeated three times independently. Statistical significance was calculated using one-way analysis of variance. *p < 0.05 vs. Sham, ^#^*p* < 0.05 vs. I/R.

In order to further clarify the effect of *L. acidophilus* ATCC 4356 on cell apoptosis in mice with renal IRI, we first detected the expression of apoptosis-related proteins in kidney tissue. The results showed that ATCC 4356 decreased the expression of pro-apoptotic factors (caspase 3, Bax, and HMGB1) and promoted the expression of anti-apoptotic factor Bcl2 at both the transcription level (Figure 4A) and translation level (Figure 4B). Next, TUNEL fluorescence experiment was applied to evaluate the level of apoptosis in kidney tissue. It could be seen from Figure 4C that ATCC 4356 reduced the apoptosis rate of renal tissue in I/R mice. The above results indicated that *L. acidophilus* ATCC 4356 inhibits cell apoptosis in mice with renal IRI.

### Effect of *Lactobacillus acidophilus* ATCC 4356 on the Gut Microbiota in Mice With Renal Ischemia–Reperfusion Injury

**Figure 5:**
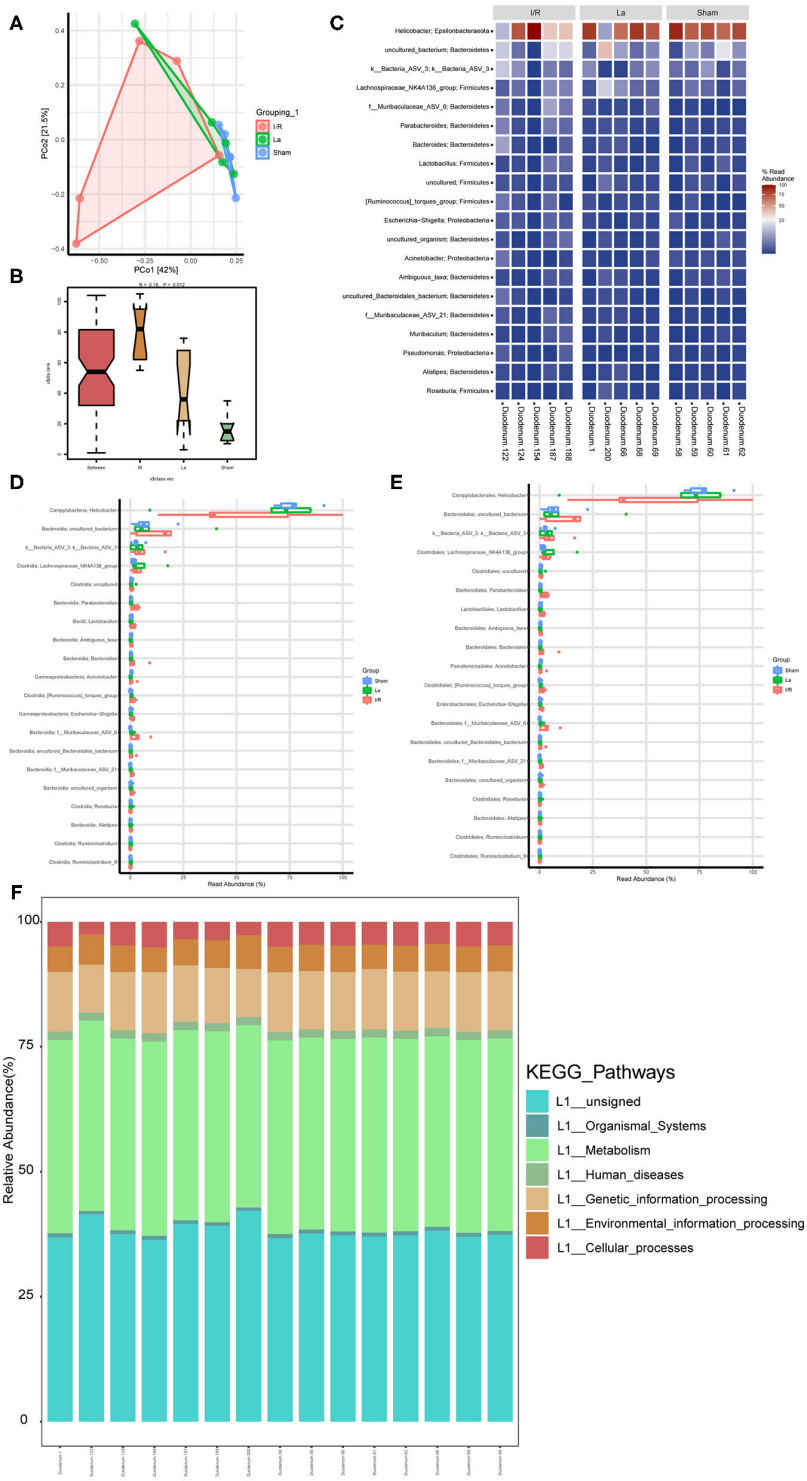
Gut microbiota composition profiles in mice with renal ischemia–reperfusion injury (IRI). **(A)** Scatter plots of principal coordinates analysis (PCoA) for gut microbiota composition to show β-diversity in Sham, ischemia/reperfusion (I/R), and La group. **(B)** Anosim analysis to evaluate the overall similarity among Sham, I/R, and La group (R > 0 and *P* < 0.05). **(C)** Heatmap to show the different expressed metabolites between Sham, I/R, and La group. **(D)** Boxplot showed the top 20 microorganisms differentially expressed among the Sham, I/R, and La groups at the class level. **(E)** Boxplot showed the top 20 microorganisms differentially expressed among the Sham, I/R, and La groups at the order level. **(F)** Kyoto Encyclopedia of Genes and Genomes (KEGG) pathway showed the gene function enrichment of each sample.

At the level of the intestinal flora, we randomly selected five mice from each group of Sham, I/R, and La group to detect the contents of the duodenum. It further showed the effect of oral *L. acidophilus* ATCC 4356 on the imbalance of intestinal flora in mice with renal IRI. The principal coordinates analysis (PCoA) was used to determined β diversity (Figure 5A). It could be seen that the gut microorganisms between the Sham group and the I/R group showed their own uniqueness, while the gut microorganisms in the La group showed a correlation with those in the Sham group. Analysis of similarities (anosim) was a statistical method that was mainly used to analyze the similarity between high-dimensional data groups (Figure 5B). Anosim analysis showed that there were significant differences among Sham, I/R, and La groups (*r* = 0.18, *P* = 0.012). The heatmap showed the top 20 differential microorganisms, and the top 3 were *Helicobacter, cultivated_bacterium*, and *k__Bacteria_ASV_3* (Figure 5C). The relative abundance of all samples at class and order levels was listed (Figures 5D,E). We further found that the relative abundance of *Helicobacter* in the Sham group was significantly higher than that in the I/R group at class and order levels. In the Sham group, the *uncultured_Bacteria* and *K__Bacteria_ASV_3* were significantly lower than those of the I/R group. At the same time, the relative abundance of these three flora in the La group was closer to that in the Sham group, which indicated that oral *L. acidophilus* ATCC 4356 effectively changed the abundance of flora. Kyoto Encyclopedia of Genes and Genomes (KEGG) annotation was performed on different groups of intestinal microorganisms (Figure 5F). The results showed that organismal systems, metabolism, human diseases, genetic information processing, environmental information processing, and cellular processes may have a certain effect on renal IRI. In conclusion, we could clearly understand that renal IRI can cause changes in intestinal microorganisms. At the same time, oral *L. acidophilus* ATCC 4356 could improve the intestinal flora imbalance caused by renal IRI.

## Discussion

Renal IRI research has always been a research hotspot of organ transplantation and general surgery (16). This study preliminarily explained the potential mechanism of ATCC 4356 to relieve renal IRI. In our study, we found that oral *L. acidophilus* ATCC 4356 alleviated the oxidative stress, inflammation, and cell apoptosis in the renal IRI mice. At the same time, ATCC 4356 regulated the homeostasis of intestinal flora in renal IRI mice. These suggested that ATCC 4356 might exert anti-oxidative stress, anti-inflammatory, and anti-apoptotic effects and improve the intestinal microbial distribution, thereby alleviating the process of renal IRI.

Decades of studies have shown that kidney tissue damage could be alleviated by reducing oxidative stress, inflammation, and cell death (17). Oral *L. acidophilus* could improve the cardiac function of mice with myocardial infarction (18). In this study, ATCC 4356 reduced the concentration of BUN and Cre in the serum of renal IRI mice. Meanwhile, H&E results showed that ATCC 4356 relieved the damage of kidney tissue in IRI mice. Oral *L. acidophilus* could protect against liver injury through its antioxidant effect, which included decreasing the expression of MDA and promoting the expression of SOD and Nrf2 (19). In renal IRI mice, we noted that ATCC 4356 increased the levels of SOD and GSH in the serum, while MDA level was reversed. This implied that ATCC 4356 played an anti-oxidative stress role in renal IRI. Nrf2/downstream antioxidant factor HO-1 (Nrf2/HO-1) axis acts an important role in anti-oxidative stress (20). On this basis, we further detected the expression of Nrf2 and HO-1 in kidney tissue at the mRNA and protein levels. The results showed that the expressions of both were upregulated with ATCC 4356 treatment in renal IRI mice. This suggested that ATCC 4356 may alleviate renal IRI through antioxidant stress.

Renal tissue damage can also be alleviated by an anti-inflammatory response. Previous studies have found that oral *L. acidophilus* could play an anti-inflammatory role in mouse colitis (21). ATCC 4356 inhibited the expression of IL-17, TNF-α, and IFN-γ, thereby mediating colon injury (22). These showed that ATCC 4356 had the potential of reducing inflammation. Our research supported this view. We found that ATCC 4356 upregulated the levels of anti-inflammatory factors (IL-4, IL-10) in renal IRI mice but downregulated the levels of pro-inflammatory factors (IL-1β, IL-8, TNF-α, and IFN-γ). In addition, ATCC 4356 exhibited an inhibitory effect on pro-apoptotic factors (caspase 3, Bax, and HMGB1) and a promotion on the anti-apoptotic factor (Bcl2). From TUNEL, it was noted that the apoptosis of kidney tissue in IRI mice was reduced *via* ATCC 4356 intervention. Therefore, we speculated that ATCC 4356 may relieve renal IRI through its influence on inflammatory signal pathway transduction. Our next work will focus on the potential signaling pathways and target cells of ATCC 4356 in renal IRI. As far as we know, changes in the structure and composition of the intestinal flora are associated with host function. Regulation of intestinal flora significantly reduced renal IRI (15). VSL#3 probiotics alleviate renal IRI by maintaining the required number of beneficial intestinal flora and inhibiting the proliferation of harmful bacteria (23). From a recent study, oral *L. acidophilus* modulated the intestinal flora structure and composition, thereby increasing the production of short-chain fatty acids (SCFAs) and reducing the number of Gram-negative bacteria to prevent chronic alcoholic liver injury in mice (24). Therefore, we suspected that ATCC 4356 alleviated renal IRI, which may be related to the intestinal flora. In this study, we further explored the effect of ATCC 4356 on the intestinal flora of renal IRI mice. The 16s DNA results showed that the relative abundance of *Helicobacter* in the Sham group was significantly higher than that in the I/R group at class and order levels. In the Sham group, the *uncultured_Bacteria* and *K__Bacteria_ASV_3* were significantly lower than those of the I/R group. And we found that the abundance of these intestinal microorganisms tended to be normal by oral *L. acidophilus* ATCC 4356, which indicated that ATCC 4356 could effectively improve the effect of IRI on the abundance of intestinal microorganisms in mice. Furthermore, it may be the reason that ATCC 4356 alleviates renal IRI, including organism system, metabolism, human diseases, genetic information processing, environmental information processing, and cellular processes.

We found that *L. acidophilus* ATCC 4356 could improve the results and composition of the intestinal flora of IRI mice, which may be a regulator of alleviating renal IRI. Intestinal flora imbalance affects the development of kidney and other diseases, which may be related to the destruction of intestinal epithelial barriers (biological barriers, physical barriers, and immune barriers) (25). Intestinal flora influences the biological barrier to participate in the process of kidney injury by secreting different metabolites, such as SCFA and trimethylamino-N-oxide (TMAO) (26). In addition, intestinal flora influences the immune barrier to participate in the process of kidney injury by targeting immune cells (27). We suspected that ATCC 4356 changes the structure of the intestinal flora, leading to changes in metabolites, and thus had a positive regulatory effect on IRI mice. This possibility and possible mechanism need further study.

Considering the limited space and budget, the mechanism study and cell model will be our next research content. Based on previous studies, bromodomain protein 4 (BRD4) inhibition alleviates renal IRI by blocking the phosphoinositide 3-kinase (PI3K)/Akt pathway to block apoptosis and oxidative stress in proximal renal tubular epithelial cells (1). *L. acidophilus* can play an anti-inflammatory role by regulating the PI3K/Akt signaling pathway (28). We will use hypoxia and reoxygenation of proximal renal tubular epithelial cells (TECs) to simulate the renal I/R model *in vivo*. Then, *L. acidophilus* ATCC 4356, PI3K agonist, or PI3K inhibitor will be used to intervene the cells. Meanwhile, qRT-PCR and Western blot will be used to detect the expression of PI3K/Akt signaling pathway, oxidative stress, inflammation, and apoptosis-related factors in cells and renal tissues. Through the above experiments, we will further investigate the mechanism of *L. acidophilus* ATCC 4356 in reducing renal IRI.

## Conclusion

In summary, *L. acidophilus* ATCC 4356 relieved renal IRI through anti-oxidative stress and anti-inflammatory response and improved the intestinal microbial distribution on renal IRI mice. This study explored the relationship between ATCC 4356 and renal IRI for the first time, which provided evidence that ATCC 4356 alleviated renal IRI. The regulation of intestinal microbiome may be a new potential mechanism for renal IRI.

## Data Availability Statement

The datasets presented in this study can be found in online repositories. The names of the repository/repositories and accession number(s) can be found at: https://www.ncbi.nlm.nih.gov/Traces/study/?acc=PRJNA703751.

## Ethics Statement

The animal study was reviewed and approved by the Animal Ethics Committee of Capital Medical University.

## Author Contributions

All authors listed have made a substantial, direct and intellectual contribution to the work, and approved it for publication.

## Conflict of Interest

The authors declare that the research was conducted in the absence of any commercial or financial relationships that could be construed as a potential conflict of interest.
